# Identification of novel *FUS* and *TARDBP* gene mutations in Chinese amyotrophic lateral sclerosis patients with HRM analysis

**DOI:** 10.18632/aging.103967

**Published:** 2020-11-05

**Authors:** Feng Wang, Shengyu Fu, Jiafan Lei, Hongchen Wu, Shugui Shi, Kangning Chen, Jun Hu, Xueqing Xu

**Affiliations:** 1 Department of Clinical Laboratory, Shenzhen Baoan Women's and Children's Hospital, Jinan University, Shenzhen, China; 2 Faculty of Health Sciences, University of Macau, Taipa, Macau, China; 3 Department of Neurology, Chunking General Hospital, Chongqing, China; 4 Department of Neurology, First Affiliated Hospital of Army Medical University, Army Medical University, Chongqing, China

**Keywords:** amyotrophic lateral sclerosis, HRM analysis, *TARDBP*, *FUS*

## Abstract

Amyotrophic lateral sclerosis (ALS) is a neurodegenerative disease characterized by progressive loss of motor neurons. More than 30 genes have been linked to ALS to date, including *FUS* and *TARDBP*, which exhibit similar roles in RNA metabolism. This study explored the use of high-resolution melting (HRM) analysis to screen for *FUS* and *TARDBP* mutation hotspot regions in 146 Chinese ALS patients, which achieved 100% detection. Two *FUS* mutations were observed in two different familial ALS probands, a missense mutation (p.R521H) and a novel splicing mutation (c.1541+1G>A). Five *TARDBP* mutations were identified in six ALS patients, including a novel 3’UTR mutation (c.*731A>G) and four missense mutations (p.G294V, p.M337V, p.G348V, and p.I383V). We found that *FUS* mutations were present in 1.4% of Chinese ALS patients, whereas *TARDBP* mutations were responsible for 4.1% of Chinese ALS cases. Here, we describe the accuracy of using highly sensitive HRM analysis to identify two novel *FUS* and *TARDBP* mutations in Chinese sporadic and familial ALS cases. Our study contributes to the further understanding of the genetic and phenotypic diversity of ALS.

## INTRODUCTION

Amyotrophic lateral sclerosis (ALS) is the most common adult-onset progressive neurodegenerative disease and is characterized by selective degeneration of upper and lower motor neurons in the brain and spinal cord [1]. The ALS median incidence rate is approximately two per 100,000 persons [2]. Median age of symptom onset is 55 years, and the average survival time is 3-5 years [3]. Approximately 10% of ALS cases are patients with familial ALS (fALS), whereas the remaining 90% are sporadic ALS (sALS) [4].

To date, more than 30 genes have been linked to ALS. However, only a few, including SOD1, C9ORF72, FUS, and TARDBP, have been identified as causative ALS genes [5, 6]. The pathogenesis of ALS remains unclear; however, one of the possible mechanisms underlying ALS is defective RNA metabolism and homeostasis [7]. Recent studies have shown that FUS and TARDBP RNA-binding proteins may contribute to ALS pathogenesis [8], and that most of these causative FUS and TARDBP mutations cluster in the C-terminus of proteins, called mutation hotspot regions [9, 10].

Sanger sequencing and next-generation sequencing for mutation screening in large samples are time consuming and expensive. In contrast, here we screen the FUS and TARDBP mutation hotspot regions in 146 ALS cases using the rapid, highly sensitive screening method, high-resolution melting (HRM) analysis. HRM analysis is a recently developed genetic analysis method for fast, high-throughput post-PCR analysis. During high-resolution melting analysis, melting curves are produced using dyes that fluoresce in the presence of double-stranded DNA (dsDNA) and specialized instruments designed to monitor fluorescence during heating; as the temperature increases, the fluorescence decreases, producing a characteristic melting profile. The shape differences in melting curves, obtained as fluorescence difference plots, are used to distinguish between mutations and controls [11, 12].

In this study, we demonstrated that highly sensitive HRM analysis can be used for screening FUS and TARDBP mutations and identified two novel mutations in Chinese sALS and fALS cases.

## RESULTS

### Sensitivity and HRM analysis

Excluding synonymous mutations and normal single-nucleotide polymorphisms, HRM analysis identified eight pathogenic mutations in 146 patients (Table 1). Similarly, targeted sequencing also detected the eight pathogenic mutations, confirming the mutation detection sensitivity of HRM analysis was 100% in this cohort. These mutations included two different FUS pathogenic mutations in two different ALS patients and five TARDBP pathogenic mutations in six ALS patients, which included two novel mutations. Therefore, FUS mutations were present in 1.4% of Chinese ALS patients, whereas TARDBP mutations were responsible for 4.1% of Chinese ALS cases.

**Table 1:** Clinical data of ALS patients carrying pathogenic mutations in FUS and TARDBP genes.

**Gene**	**Nucleotide change**	**Amino acid change**	**Type of mutation**	**Onset; age of onset;gender**	**Disease duration**	**ALS family history**	**References**
FUS	c.1541+1G>A	-	splicing	Bulbar; 51; M	1 y	Yes	This study
FUS	c.1562G>A	p. R521H	missense	Spinal; 41; M	3 y	Yes	[14]
TARDBP	c.881G>T	p. G294V	missense	Bulbar, Spinal; 61; M	0.5 y	No	[15, 16]
				Bulbar; 53; M	1.5+y	No
TARDBP	c.1009A>G	p. M337V	missense	Spinal; 58; M	1+y	Yes	[17]
TARDBP	c.1043G>T	p. G348V	missense	Spinal; 24; M	10+y	No	[18]
TARDBP	c.1147A>G	p. I383V	missense	Spinal; 45; F	3 y	No	[19]
TARDBP	c. *731A>G	-	3’UTR	-;N/A;M	-	No	This study

The symbol + indicates disease in progress.

Key: ALS, amyotrophic lateral sclerosis; F, female; M, male; N/A, information not available

### FUS mutations and clinical characteristics

**Figure 1:**
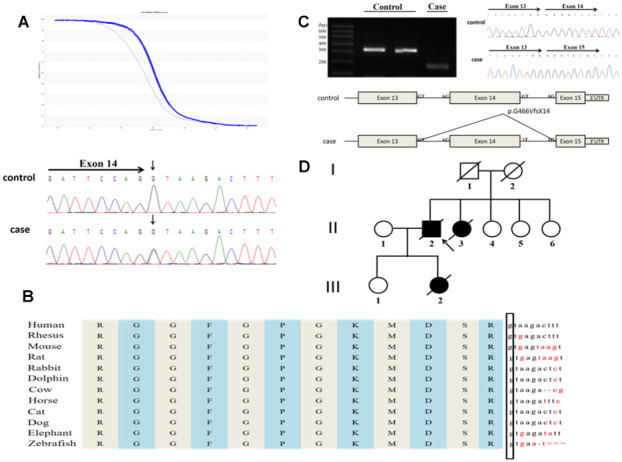
**A novel splicing mutation, c.1541+1G>A in the FUS gene identified in a Chinese ALS patient.** (**A**) The novel splicing mutation was identified by HRM analysis and direct sequencing. (**B**) The evolutionary conservation of the splicing mutation c.1541+1G>A are shown. (**C**) Minigene splicing analysis of the novel splice mutation. (**D**) Pedigree of the family. An arrowhead indicates the proband.

Figure 1A shows the detected novel splicing mutation, c.1541+1G>A (Figure 1A), located at the splice donator site of the FUS intron 14. The guanine of the wild-type donator site is highly conserved across species (Figure 1B). A functional splicing reporter minigene assay confirmed the novel splicing mutation result in a frameshift, leading to truncated FUS protein p.G466VfsX14 (Figure 1C).

The novel splicing mutation was identified in a 51-year-old male patient whose daughter previously died from ALS. Initially, he presented with neck weakness and dysphagia. After 5 months, lower right limb weakness emerged and gradually increased without cognitive impairment. The patient died 13 months after onset of symptoms. A 20-year-old female patient, the daughter of the 51-year-old patient, experienced lower right limb weakness, which quickly developed in all four extremities within 1 year. After 12 months, she died of respiratory failure. Figure 1D shows the same mutation was detected in the sister of the 51-year-old patient’s sister and shared a similar clinical phenotype; the disease duration of this patient was only 8 months.

**Figure 2:**
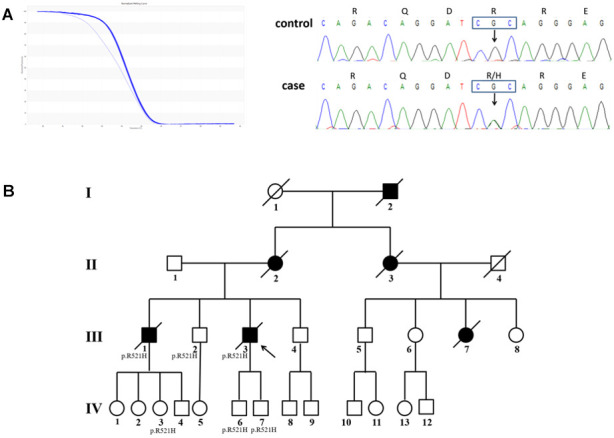
**The pedigree with FUS mutation p. R521H.** (**A**) Results of HRM analysis and direct sequencing of patients. (**B**) Pedigree of the family. An arrowhead indicates the proband. Patients III2, IV3, IV6, and IV7 shared the same mutation, but they did not appear as ALS phenotypes until now.

A heterozygous missense substitution, c.1562G>A (p. R521H, Figure 2A), was identified in an index fALS patient who began suffering from progressive weakness in the upper-right limb at 41 years of age. Twelve months later, the weakness developed in other limbs. Then, he presented with dysarthria and dysphagia after 13 months and without cognitive impairment. The patient died 3 years after onset of symptoms. The patient’s brother who shared the same mutation (patient III1) also had a similar clinical phenotype and disease duration. Patient III2, as well as several family members of patients III1 and III3, also share the mutation; however, they did not show any clinical characteristics of ALS until now (Figure 2B).

### TARDBP mutations and clinical characteristics

**Figure 3:**
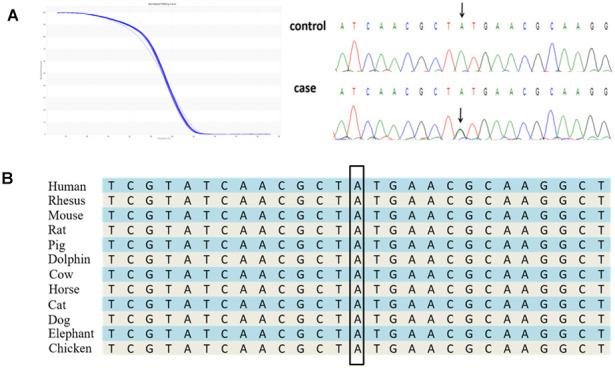
A novel 3′UTR mutation, c. *731A>G in the TARDBP gene identified in a Chinese ALS patient. (**A**) The novel 3′UTR mutation was identified by HRM analysis and direct sequencing. (**B**) The evolutionary conservation of the TARDBP 3′UTR mutation c. *731A>G.

Five TARDBP mutations were observed in six unrelated ALS patients (Figure 3A), which included a novel 3′UTR mutation (c. *731A>G) and four known causative mutations (p.G294V, p.G348V, p.M337V, and p.I383V). According to the UCSC Genome Browser, the TARDBP 3′-UTR site c. *731 is highly conserved through the evolutionary spectrum from human to chicken (Figure 3B), supporting its functional importance. MicroRNA (miRNA) binding searches of TARDBP through the TargetScanHuman database showed binding of miR-376a directly to the 3′UTR variant region. The novel c. *731A>G TARDBP mutation was expected to interfere with the normal binding of miR-376, and then disorder the expression of TARDBP mRNA. However, we sequenced this region of the patient’s parents and confirmed this was a de novo mutation. In addition, the mutation was not observed in available variation databases, including mutation database and SNP databases such as the National Center for Biotechnology Information (NCBI) Single Nucleotide Polymorphism Database (http://www.ncbi.nlm.nih.gov/projects/SNP/), Exome Aggregation Consortium (ExAC) database (http://exac.broadinstitute.org), and ALS Online Genetics Database (http://alsod.iop.kcl.ac.uk/) [13]. According to American College of Medical Genetics and Genomics guidelines, it meets de novo criteria PS2 (in a patient with the disease and no family history), PM2 (absent from controls or at extremely low frequency if recessive in Exome Sequencing Project, 1000 Genomes Project, or Exome Aggregation Consortium), and PP4 (patient’s phenotype or family history is highly specific for a disease with a single genetic etiology) for classifying pathogenic variants. We suggest this mutation is a “likely pathogenic” for the previously stated reason.

The TARDBP p.G294V mutation is located in the sixth exon of the gene and was present in two unrelated male sALS patients. The patient began undergoing dysarthria with weakness in the upper right limb. Five months later, the weakness developed in the upper left limb, and dysphagia occasionally occurred. He died 6 months after onset of symptoms. At age 53, the second patient experienced symptoms primarily characterized by dysarthria. Neither ALS patient carrying the p.G294V mutation showed signs of cognitive dysfunction.

The p.G348V mutation was identified in a 24-year-old juvenile-onset male patient who reported no family history of ALS. During the initial onset of symptoms, the patient showed slight fasciculation in the upper right limb. During the next 3 years, the fasciculation was progressive and developed in the upper left limb. The MRI images show that the anisotropy of cortical spinal tract scores in the left midbrain was indicative of white matter degeneration.

To date, fasciculation and muscle atrophy have been observed in all extremities.

A 45-year-old female patient carrying the p.I383V mutation presented with progressive weakness in the upper limb, and electromyography results suggested spinal anterior of the lower cervical was injured. After 3 years, the patient died of respiratory failure.

## DISCUSSION

Following rapid development of molecular genetics, many genes have been identified with ALS pathogenesis. Although the exact mechanism for ALS is still unknown, several hypotheses include defective RNA metabolism, glutamate excitotoxicity, disruption of membrane trafficking, endoplasmic reticulum stress, mitochondrial dysfunction, and protein misfolding and aggregation [20]. Some mutations in FUS and TARDBP genes, which both play an important role in mRNA transport, axonal maintenance, and motor neuron development, have been reported as causative ALS mutations for disturbing RNA homeostasis [4, 9, 21]. Interestingly, because the vast majority of these causative mutations in FUS and TARDBP cluster in the C-terminus of the proteins, also known as mutation hotspot regions, [9, 10] this suggested we could focus on mutation detection at the mutational hotspot regions, instead of the whole gene.

Although Sanger sequencing and next-generation sequencing are two of most common mutation detection methods for large-scale genomics sequencing samples, they can be cost prohibitive and slow. In this study, we first identified the FUS and TARDBP mutations in Chinese ALS patients by the fast and highly sensitive screening method, HRM analysis.

Using HRM analysis, we successfully identified five known possible causative mutations, and two novel mutations. The mutation frequency was 1.4% for FUS, and 4.1% for TARDBP in Chinese ALS patients. However, the frequency of our study is inconsistent with previously reported results, [22] which may be due to small sample size and population difference.

We identified two FUS pathogenic mutations in this study. Previous reports identified mutation p.R521H as the highest FUS mutation frequency, accounting for 30%, and experimental results show that the FUS R521H missense mutation could lead to aberrant trafficking and retention of the mutant FUS protein in the cytoplasm [14, 23, 24]. Interestingly, several relatives and a sibling of the proband also have the p.R521H mutation; however, they did not present with clinically relevant ALS symptoms, which may be related to age or the presence of a recessive inheritance pattern [14, 25].

A novel splicing mutation, c.1541+1G>A, the third splicing mutation linked to ALS in FUS gene [26, 27], produced a truncated FUS protein, p.G466VfsX14, eliminating the nuclear localization sequence (NLS), where most previously published mutations are located [9], and then impair its nuclear import. Previous results show that overexpression of cytoplasmic FUS with deleted NLS results in recapitulation of ALS-like motor neuron abnormalities in mice, [28] indicating this mutation is significant. Furthermore, mutation of a truncated protein lacking the NLS was reported to cause juvenile ALS with rapid disease progression [29, 30], which was consistent with the clinical symptoms in our study. Subsequent research is still needed to study the function of the mutation. The novel splicing mutation we found confirms the C-terminal NLS of the FUS gene plays a key role in ALS pathology.

We identified five TARDBP mutations in our ALS cohort, four of which have previously been reported as causative mutations. The p.G294V mutation was previously identified in a sALS patient from Italy and in a fALS patient from Australia, and was predicted to disrupt the glycine-rich domain in the C-terminus, which plays a role in RNA binding and is required for the exon-skipping activity of TARDBP [15, 31]. Previous reports showed the percentages of cells with positive nuclear immunostaining were significantly lower in patients with TARDBP p.G294V mutations than in controls, indicating its importance [16]. In this study, we identified p.G294V mutations in two unrelated sALS patients, for whom the onset of symptoms is characterized by dysarthria. To our knowledge, this is the first Asian population study for this mutation. The p.G348V mutation, which increases the amount of TARDBP protein at the cellular level, was detected in ALS patients from various populations [18, 32–34]. However, there are some differences for the ALS clinical symptoms between the western and Chinese populations. The age of onset for Chinese ALS cases were much younger than previously published western cases. Additionally, the mutation carriers in the Chinese population experienced slower disease progression and longer survival time [34]. Our study confirmed these differences. The third mutation we identified, p.I383V, has been detected in fALS patients from the United States, Turkey, and Taiwan. However, the onset and progression of the disease for the carrier p.I383V are different [19, 35–37]. In our study, the mutation p.I383V was identified from a sALS woman who suffered progressive weakness in the upper limb at the age of 45, and died 3 years later.

The final mutation we identified, the novel 3'UTR mutation c. *731A>G, was a de novo and was not observed in the parents of patients. Additionally, it has not be reported in any mutation databases, and the site c. *731 is highly conserved. According to the software, the mutation may affect the combination of mir-376a for TARDBP and then lead to increasing mRNA levels, which may accelerate disease progression by inducing the formation of cytoplasmic inclusions and/or the dysregulation of RNA metabolism [38]. In addition, a previous study reported a similar 3'UTR mutation linked to ALS [39]. According to the guideline of ACMG, we suggested c. *731A>G as a likely pathogenic mutation for ALS. Unfortunately, we did not have access to detailed medical records and fresh blood samples.

In conclusion, to our knowledge, this is the first time that HRM analysis was used to identify FUS and TARDBP mutations in Chinese patients with ALS, demonstrating its high sensitivity and accuracy. Our data suggests that HRM analysis is a fast and easy method for mutation screening in large samples. Most important, however, we identified two novel pathogenic ALS mutations, which further expands the ALS-related gene mutation spectrum, and provides data for subsequent studies. We also investigated the frequency of FUS and TARDBP mutations in the Chinese population.

## MATERIALS AND METHODS

### Subjects

The study included 146 patients with sALS and nine fALS index cases (male: 62.3%; age at onset: 47.9 ± 11.7 years). All patients were recruited from the Department of Neurology, Southwest Hospital, Third Military Medical University, Chongqing between 2012 and 2017. All patients were Han Chinese, and the patients met the El Escorial criteria for ALS diagnosis [40]. Family history was considered positive if the patient had at least one affected relative within three generations [41]. All participants provided informed consent before blood donation. Protocols were in accordance with the ethical standards of the responsible committee on human experimentation and the Helsinki Declaration of 1964 and approved by the institutional ethics committee of the Hospital (equivalent to an Institutional Review Board). This study was carried out in accordance with the approved guidelines. Written informed consent was obtained from all participants. All patients were tested for the C9orf72 GGGGCC expanding mutation with repeat-primed PCR, together with proper positive control and negative control. No C9orf72 GGGGCC expanding mutation was detected in this cohort.

### Screening TARDBP and FUS gene mutations by PCR-HRM

We designed PCR primers for HRM analysis to screen all of the mutation hotspot regions containing OMIM Allelic Variants mutations related to ALS (from the UCSC Genome Browser) of FUS (NM_004960.3) and TARDBP (NM_007375.3). Table 2 shows the amplified primer sequence and the length of each expected fragment.

**Table 2:** PCR primer sequences of FUS and TARDBP gene and size of amplified fragment.

**Gene**	**Primer**	**Primer sequence**	**Fragment size**
FUS	1	5′-GAGGGTAACACTGGGTACAGGAC-3′	160
		5′-GGCTTGGAGAGGCTGGTAAC-3′	
	2	5′-GGTTATGGCAATCAAGACCAGA-3′	149
		5′-CTGGGTTCATAGCCACCACT-3′	
	3	5′- GCTATGATCGAGGCGGCTA -3′	158
		5′-TGGCCTCTGTTCAACTGCTC -3′	
TARDBP	1	5′-CGGAATATGAAACACAAGTGAAAG-3′	82
		5′-GAATTAGGAAGTTTGCAGTCACAC-3′	
	2	5′-CCGAACCTAAGCACAATAGCA-3′	156
		5′-TCATCCCACCACCCATATTACT-3′	
	3	5′-AATATGGGTGGTGGGATGAAC -3′	160
		5′-TGTTGCCTTGGTTTTGGTTATT-3′	
	4	5′-AATAACCAAAACCAAGGCAACAT-3′	157
		5′-TTGAGCCAAAGCCTCCATTA- 3′	
	5	5′-GGGTTGTGGTTGGTTGGTATAG-3′	130
		5′-CTGCTGAATATACTCCACACTGAAC-3′	
	6	5′-GCCATAGGAATACTGTCTACATGCT-3′	152
		5′-CCATATCACAGCCTTGCGTT-3′	

Genomic DNA was extracted from peripheral blood leukocytes using standard methods.

PCR-HRM was performed with Eco Real-Time PCR System (Illumina) in 10-μL reaction mixtures comprising 20 ng of DNA, 1×PCR buffer, 0.2 mM dNTP, Eva Green (Biotium),1U of Taq polymerase, and 0.25 μM each forward and reverse primers. Initial denaturation was performed at 95°C for 5 min, followed by 50 cycles of 95°C for 20 sec, 60°C for 20 sec, and 72°C for 20 sec. The melting curves were obtained under the following conditions: 95°C for 15 sec, 55°C for 15 sec, and with a ramping rate of +0.2°C/sec to 95°C.

### Direct DNA sequencing and bioinformatics

To confirmed the genotype of mutations and the specificity of HRM analysis, All PCR products showing an HRM aberrant pattern were analyzed with direct DNA sequencing on a PRISM 377 full-automatic sequencing analyzer (ABI).

When the DNA sequencing result was determined, we first confirmed whether there was a mutation aimed to detect the specificity of HRM analysis. Then, we ensured it was a novel mutation using the HGMD database (http://www.hgmd.cf.ac.uk/ac) and the dbSNP database (http://www.ncbi.nlm.nih.gov/snp). Finally, to evaluate the potential functional effect of the novel splicing mutation and 3'UTR site mutation, we used the UCSC Genome Browser (http://genome.ucsc.edu/), Target (http://www.targetscan.org/vert_71), Human Splicing Finder (http://www.umd.be/HSF3/). To identify known variations, we used ALS Online Genetics Database (http://alsod.iop.kcl.ac.uk/) (13), the NCBI Database (http://www.ncbi.nlm.nih.gov), and Exome Aggregation Consortium (ExAC) database (http://exac.broadinstitute.org).

### Targeted sequencing

To verify the sensitivity of HRM analysis, we sequenced all exons of the FUS and TARDBP gene in 146 patients by Illumina Hiseq 3000 system. The target coverage is 98% with an average depth of 100X. All nonsynonymous variants detected were filtered.

### Functional splicing reporter minigene assay

Because of the lack of simple RNA, we used splicing reporter minigene assay to confirm the functional effect of the novel splicing mutation. The minigene was constructed by PCR amplification of mutant genomic DNA sequences as previously described [42]. The amplified sequences included exons 13 through 3’UTR of the FUS gene. FUS minigene products were digested with BamH I and Xho I (Thermo) and cloned into the pCDNA3.1(+) reporter vector. The resulting constructs were transfected into U87 cells. Total cellular RNA was extracted after 24 hours, followed by RT-PCR analysis.

## References

[1] Kiernan MC, Vucic S, Cheah BC, Turner MR, Eisen A, Hardiman O, Burrell JR, Zoing MC. Amyotrophic lateral sclerosis. Lancet. 2011; 377:942–55. 10.1016/S0140-6736(10)61156-7 21296405

[2] Chiò A, Logroscino G, Traynor BJ, Collins J, Simeone JC, Goldstein LA, White LA. Global epidemiology of amyotrophic lateral sclerosis: a systematic review of the published literature. Neuroepidemiology. 2013; 41:118–30. 10.1159/000351153 23860588 PMC4049265

[3] Qureshi M, Schoenfeld DA, Paliwal Y, Shui A, Cudkowicz ME. The natural history of ALS is changing: improved survival. Amyotroph Lateral Scler. 2009; 10:324–31. 10.3109/17482960903009054 19922119

[4] Renton AE, Chiò A, Traynor BJ. State of play in amyotrophic lateral sclerosis genetics. Nat Neurosci. 2014; 17:17–23. 10.1038/nn.3584 24369373 PMC4544832

[5] Freischmidt A, Wieland T, Richter B, Ruf W, Schaeffer V, Müller K, Marroquin N, Nordin F, Hübers A, Weydt P, Pinto S, Press R, Millecamps S, et al. Haploinsufficiency of TBK1 causes familial ALS and fronto-temporal dementia. Nat Neurosci. 2015; 18:631–36. 10.1038/nn.4000 25803835

[6] Vajda A, McLaughlin RL, Heverin M, Thorpe O, Abrahams S, Al-Chalabi A, Hardiman O. Genetic testing in ALS: a survey of current practices. Neurology. 2017; 88:991–99. 10.1212/WNL.0000000000003686 28159885 PMC5333513

[7] Taylor JP, Brown RH Jr, Cleveland DW. Decoding ALS: from genes to mechanism. Nature. 2016; 539:197–206. 10.1038/nature20413 27830784 PMC5585017

[8] Shang Y, Huang EJ. Mechanisms of FUS mutations in familial amyotrophic lateral sclerosis. Brain Res. 2016; 1647:65–78. 10.1016/j.brainres.2016.03.036 27033831 PMC5003642

[9] Lattante S, Rouleau GA, Kabashi E. TARDBP and FUS mutations associated with amyotrophic lateral sclerosis: summary and update. Hum Mutat. 2013; 34:812–26. 10.1002/humu.22319 23559573

[10] Drepper C, Herrmann T, Wessig C, Beck M, Sendtner M. C-terminal FUS/TLS mutations in familial and sporadic ALS in Germany. Neurobiol Aging. 2011; 32:548.e1–4. 10.1016/j.neurobiolaging.2009.11.017 20018407

[11] Wang J, Ren X, Bai X, Zhang T, Wang Y, Li K, Li G. Identification of gene mutation in patients with osteogenesis imperfect using high resolution melting analysis. Sci Rep. 2015; 5:13468. 10.1038/srep13468 26307460 PMC4549685

[12] Montgomery J, Wittwer CT, Palais R, Zhou L. Simultaneous mutation scanning and genotyping by high-resolution DNA melting analysis. Nat Protoc. 2007; 2:59–66. 10.1038/nprot.2007.10 17401339

[13] Abel O, Powell JF, Andersen PM, Al-Chalabi A. ALSoD: a user-friendly online bioinformatics tool for amyotrophic lateral sclerosis genetics. Hum Mutat. 2012; 33:1345–51. 10.1002/humu.22157 22753137

[14] Kwiatkowski TJ Jr, Bosco DA, Leclerc AL, Tamrazian E, Vanderburg CR, Russ C, Davis A, Gilchrist J, Kasarskis EJ, Munsat T, Valdmanis P, Rouleau GA, Hosler BA, et al. Mutations in the FUS/TLS gene on chromosome 16 cause familial amyotrophic lateral sclerosis. Science. 2009; 323:1205–08. 10.1126/science.1166066 19251627

[15] Del Bo R, Ghezzi S, Corti S, Pandolfo M, Ranieri M, Santoro D, Ghione I, Prelle A, Orsetti V, Mancuso M, Sorarù G, Briani C, Angelini C, et al. TARDBP (TDP-43) sequence analysis in patients with familial and sporadic ALS: identification of two novel mutations. Eur J Neurol. 2009; 16:727–32. 10.1111/j.1468-1331.2009.02574.x 19236453

[16] Sabatelli M, Zollino M, Conte A, Del Grande A, Marangi G, Lucchini M, Mirabella M, Romano A, Piacentini R, Bisogni G, Lattante S, Luigetti M, Rossini PM, Moncada A. Primary fibroblasts cultures reveal TDP-43 abnormalities in amyotrophic lateral sclerosis patients with and without SOD1 mutations. Neurobiol Aging. 2015; 36:2005.e5–13. 10.1016/j.neurobiolaging.2015.02.009 25792239

[17] Sreedharan J, Blair IP, Tripathi VB, Hu X, Vance C, Rogelj B, Ackerley S, Durnall JC, Williams KL, Buratti E, Baralle F, de Belleroche J, Mitchell JD, et al. TDP-43 mutations in familial and sporadic amyotrophic lateral sclerosis. Science. 2008; 319:1668–72. 10.1126/science.1154584 18309045 PMC7116650

[18] Kirby J, Goodall EF, Smith W, Highley JR, Masanzu R, Hartley JA, Hibberd R, Hollinger HC, Wharton SB, Morrison KE, Ince PG, McDermott CJ, Shaw PJ. Broad clinical phenotypes associated with TAR-DNA binding protein (TARDBP) mutations in amyotrophic lateral sclerosis. Neurogenetics. 2010; 11:217–25. 10.1007/s10048-009-0218-9 19760257

[19] Rutherford NJ, Zhang YJ, Baker M, Gass JM, Finch NA, Xu YF, Stewart H, Kelley BJ, Kuntz K, Crook RJ, Sreedharan J, Vance C, Sorenson E, et al. Novel mutations in TARDBP (TDP-43) in patients with familial amyotrophic lateral sclerosis. PLoS Genet. 2008; 4:e1000193. 10.1371/journal.pgen.1000193 18802454 PMC2527686

[20] Peters OM, Ghasemi M, Brown RH Jr. Emerging mechanisms of molecular pathology in ALS. J Clin Invest. 2015; 125:2548. 10.1172/JCI82693 26030230 PMC4518693

[21] Nolan M, Talbot K, Ansorge O. Pathogenesis of FUS-associated ALS and FTD: insights from rodent models. Acta Neuropathol Commun. 2016; 4:99. 10.1186/s40478-016-0358-8 27600654 PMC5011941

[22] Zou ZY, Zhou ZR, Che CH, Liu CY, He RL, Huang HP. Genetic epidemiology of amyotrophic lateral sclerosis: a systematic review and meta-analysis. J Neurol Neurosurg Psychiatry. 2017; 88:540–49. 10.1136/jnnp-2016-315018 28057713

[23] Dormann D, Rodde R, Edbauer D, Bentmann E, Fischer I, Hruscha A, Than ME, Mackenzie IR, Capell A, Schmid B, Neumann M, Haass C. ALS-associated fused in sarcoma (FUS) mutations disrupt transportin-mediated nuclear import. EMBO J. 2010; 29:2841–57. 10.1038/emboj.2010.143 20606625 PMC2924641

[24] Bosco DA, Lemay N, Ko HK, Zhou H, Burke C, Kwiatkowski TJ Jr, Sapp P, McKenna-Yasek D, Brown RH Jr, Hayward LJ. Mutant FUS proteins that cause amyotrophic lateral sclerosis incorporate into stress granules. Hum Mol Genet. 2010; 19:4160–75. 10.1093/hmg/ddq335 20699327 PMC2981014

[25] Bertolin C, D’Ascenzo C, Querin G, Gaiani A, Boaretto F, Salvoro C, Vazza G, Angelini C, Cagnin A, Pegoraro E, Sorarù G, Mostacciuolo ML. Improving the knowledge of amyotrophic lateral sclerosis genetics: novel SOD1 and FUS variants. Neurobiol Aging. 2014; 35:1212.e7–10. 10.1016/j.neurobiolaging.2013.10.093 24325798

[26] DeJesus-Hernandez M, Kocerha J, Finch N, Crook R, Baker M, Desaro P, Johnston A, Rutherford N, Wojtas A, Kennelly K, Wszolek ZK, Graff-Radford N, Boylan K, Rademakers R. De novo truncating FUS gene mutation as a cause of sporadic amyotrophic lateral sclerosis. Hum Mutat. 2010; 31:E1377–89. 10.1002/humu.21241 20232451 PMC2922682

[27] Belzil VV, St-Onge J, Daoud H, Desjarlais A, Bouchard JP, Dupré N, Camu W, Dion PA, Rouleau GA. Identification of a FUS splicing mutation in a large family with amyotrophic lateral sclerosis. J Hum Genet. 2011; 56:247–49. 10.1038/jhg.2010.162 21160488

[28] Shiihashi G, Ito D, Yagi T, Nihei Y, Ebine T, Suzuki N. Mislocated FUS is sufficient for gain-of-toxic-function amyotrophic lateral sclerosis phenotypes in mice. Brain. 2016; 139:2380–94. 10.1093/brain/aww161 27368346

[29] Ito D, Yagi T, Ikawa M, Suzuki N. Characterization of inclusion bodies with cytoprotective properties formed by seipinopathy-linked mutant seipin. Hum Mol Genet. 2012; 21:635–46. 10.1093/hmg/ddr497 22045697

[30] Hou L, Jiao B, Xiao T, Zhou L, Zhou Z, Du J, Yan X, Wang J, Tang B, Shen L. Screening of SOD1, FUS and TARDBP genes in patients with amyotrophic lateral sclerosis in central-southern China. Sci Rep. 2016; 6:32478. 10.1038/srep32478 27604643 PMC5015023

[31] Williams KL, Durnall JC, Thoeng AD, Warraich ST, Nicholson GA, Blair IP. A novel TARDBP mutation in an Australian amyotrophic lateral sclerosis kindred. J Neurol Neurosurg Psychiatry. 2009; 80:1286–88. 10.1136/jnnp.2008.163261 19864664

[32] Brown JA, Min J, Staropoli JF, Collin E, Bi S, Feng X, Barone R, Cao Y, O’Malley L, Xin W, Mullen TE, Sims KB. SOD1, ANG, TARDBP and FUS mutations in amyotrophic lateral sclerosis: a United States clinical testing lab experience. Amyotroph Lateral Scler. 2012; 13:217–22. 10.3109/17482968.2011.643899 22292843

[33] Budini M, Romano V, Avendaño-Vázquez SE, Bembich S, Buratti E, Baralle FE. Role of selected mutations in the Q/N rich region of TDP-43 in EGFP-12xQ/n-induced aggregate formation. Brain Res. 2012; 1462:139–50. 10.1016/j.brainres.2012.02.031 22406069

[34] Zou ZY, Peng Y, Wang XN, Liu MS, Li XG, Cui LY. Screening of the TARDBP gene in familial and sporadic amyotrophic lateral sclerosis patients of Chinese origin. Neurobiol Aging. 2012; 33:2229.e11–18. 10.1016/j.neurobiolaging.2012.03.014 22575358

[35] Gendron TF, Rademakers R, Petrucelli L. TARDBP mutation analysis in TDP-43 proteinopathies and deciphering the toxicity of mutant TDP-43. J Alzheimers Dis. 2013 (Suppl 1); 33:S35–45. 10.3233/JAD-2012-129036 22751173 PMC3532959

[36] Cheng YW, Lee MJ, Chen TF, Cheng TW, Lai YM, Hua MS, Chiu MJ. A single nucleotide TDP-43 mutation within a Taiwanese family: a multifaceted demon. Amyotroph Lateral Scler Frontotemporal Degener. 2016; 17:292–94. 10.3109/21678421.2015.1111905 26581115

[37] Özoğuz A, Uyan Ö, Birdal G, Iskender C, Kartal E, Lahut S, Ömür Ö, Agim ZS, Eken AG, Sen NE, Kavak P, Saygı C, Sapp PC, et al. The distinct genetic pattern of ALS in Turkey and novel mutations. Neurobiol Aging. 2015; 36:1764.e9–18. 10.1016/j.neurobiolaging.2014.12.032 25681989 PMC6591733

[38] Koyama A, Sugai A, Kato T, Ishihara T, Shiga A, Toyoshima Y, Koyama M, Konno T, Hirokawa S, Yokoseki A, Nishizawa M, Kakita A, Takahashi H, Onodera O. Increased cytoplasmic TARDBP mRNA in affected spinal motor neurons in ALS caused by abnormal autoregulation of TDP-43. Nucleic Acids Res. 2016; 44:5820–36. 10.1093/nar/gkw499 27257061 PMC4937342

[39] Gitcho MA, Bigio EH, Mishra M, Johnson N, Weintraub S, Mesulam M, Rademakers R, Chakraverty S, Cruchaga C, Morris JC, Goate AM, Cairns NJ. TARDBP 3'-UTR variant in autopsy-confirmed frontotemporal lobar degeneration with TDP-43 proteinopathy. Acta Neuropathol. 2009; 118:633–45. 10.1007/s00401-009-0571-7 19618195 PMC2783457

[40] Brooks BR, Miller RG, Swash M, Munsat TL, and World Federation of Neurology Research Group on Motor Neuron Diseases. El escorial revisited: revised criteria for the diagnosis of amyotrophic lateral sclerosis. Amyotroph Lateral Scler Other Motor Neuron Disord. 2000; 1:293–99. 10.1080/146608200300079536 11464847

[41] Tsai PC, Liu YC, Lin KP, Liu YT, Liao YC, Hsiao CT, Soong BW, Yip PK, Lee YC. Mutational analysis of TBK1 in Taiwanese patients with amyotrophic lateral sclerosis. Neurobiol Aging. 2016; 40:191.e11–16. 10.1016/j.neurobiolaging.2015.12.022 26804609

[42] Cooper TA. Use of minigene systems to dissect alternative splicing elements. Methods. 2005; 37:331–40. 10.1016/j.ymeth.2005.07.015 16314262

